# Coherent acoustic phonons in a coupled hexagonal boron nitride–graphite heterostructure

**DOI:** 10.1063/4.0000228

**Published:** 2024-02-14

**Authors:** Arne Ungeheuer, Nora Bach, Mashood T. Mir, Ahmed S. Hassanien, Lukas Nöding, Thomas Baumert, Sascha Schäfer, Arne Senftleben

**Affiliations:** 1 Institute of Physics, University of Kassel, 34132 Kassel, Germany; 2 Institute of Physics, University of Oldenburg, 26129 Oldenburg, Germany

## Abstract

Femtosecond optically excited coherent acoustic phonon modes (CAPs) are investigated in a free-standing van der Waals heterostructure composed of a 20-nm transparent hexagonal boron nitride (hBN) and a 42-nm opaque graphite layer. Employing ultrafast electron diffraction, which allows for the independent evaluation of strain dynamics in the constituent material layers, three different CAP modes are identified within the bilayer stack after the optical excitation of the graphite layer. An analytical model is used to discuss the creation of individual CAP modes. Furthermore, their excitation mechanisms in the heterostructure are inferred from the relative phases of these modes by comparison with a numerical linear-chain model. The results support an ultrafast heat transfer mechanism from graphite to the hBN lattice system, which is important to consider when using this material combination in devices.

## INTRODUCTION

The study of photoexcited coherent strain waves in bulk materials, thin-films, and nanostructures has developed into a broad field of research over the last decades, leading to many exciting findings in both fundamental research and applications. For instance, coherent strain waves serve as a tool to probe buried nanostructures by optically generating a strain wave packet at the surface of a specimen and inferring structural information from the signal that is reflected from a buried topography.^1,2^ In contrast to propagating strain waves, different structural dynamics are observed in thin-films when the film thickness is decreased to the nano-regime, and the out-of-plane spatial extent of the photoexcited strain-region becomes comparable to the thickness of the specimen. This confinement results in discrete standing wave solutions for the atomic displacement and strain fields. These solutions depend on the specific boundary conditions imposed on the system, e.g., different types of modes are excited depending on whether the nanomembrane is free-standing or has been prepared on a supporting substrate. The oscillatory out-of-plane compression and expansion, termed CAP breathing mode, has been investigated in optically excited nanofilms for several classes of materials, e.g., in semiconductors,^3,4^ metals,^5–8^ and more recently also in thin van der Waals materials.^9–12^ Since the first isolation of mechanically exfoliated graphene in 2004,^13,14^ van der Waals materials have become of large interest due to often unique anisotropic electrical, thermal, and optical properties,^15–17^ which is why they have continued to generate large interest in numerous research areas.

The possibility to combine different van der Waals materials by vertical stacking of exfoliated crystal films has furthermore allowed for the study of van der Waals heterostructures and twisted homojunctions, some of which display completely new or altered physical properties compared to the isolated materials.^18–20^ Coherent strain waves in van der Waals bilayer systems became first accessible by all optical methods applied to InSe–hBN heterostructures and InSe–InSe homojunctions prepared on a sapphire substrate.^21^ The authors demonstrated perfect elastic coupling between the stacked van der Waals materials, but a weaker coupling of the van der Waals layers to the substrate.

In this work, we extend the study of coherent strain waves in stacked van der Waals systems to ultrafast electron diffraction (UED) measurements on a quasi-free-standing hBN–graphite heterostructure. The combination of hBN and graphite is particularly interesting because of the intricate pathways for CAP excitation: Optical excitation of carriers in graphite is followed by fast relaxation to a narrow set of strongly coupled optical phonons (SCOPs) on the sub-picosecond timescale,^22,23^ resulting in a long-lived non-thermal phonon population that subsequently couples to lower frequency acoustic phonons on a 10-ps timescale.^10,24^ Since hBN is a wide-bandgap semiconductor, direct excitation of the electronic system in the hBN layer can be excluded for the optical wavelength and fluence used in the experiment. However, recent studies have reported evidence for an ultrafast heat transfer mechanism from either the graphite's electronic system or the SCOPs to the hBN lattice.^25,26^ Since the photogenerated stress, and therefore the spectrum of excited CAP modes, is directly related to the spatiotemporal temperature distribution in the heterostructure after laser excitation,^27–29^ the question arises which CAP excitation mechanisms are at work for the heterogeneous system considered here. To shed further light on this, we numerically modeled the transient stress for different excitation scenarios employing a one-dimensional linear-chain model and extract the dominant excitation mechanism from comparison of the CAP mode's amplitudes and phases with our experimental results. In the discussion of the experimental data, we take into account the sensitivity of the UED measurement technique to different CAP modes as well as their excitation strength based on the atomic displacement and stress fields corresponding to the observed CAP modes. Two excitation scenarios are considered in which only the optically excited graphite layer is assumed to contribute to stress generation (with different contributions of deformation potential and thermoelastic stress), and one scenario is analyzed in which a possible additional thermoelastic stress contribution in the hBN layer is included.

## METHODS

### Ultrafast electron diffraction

**FIG. 1.**
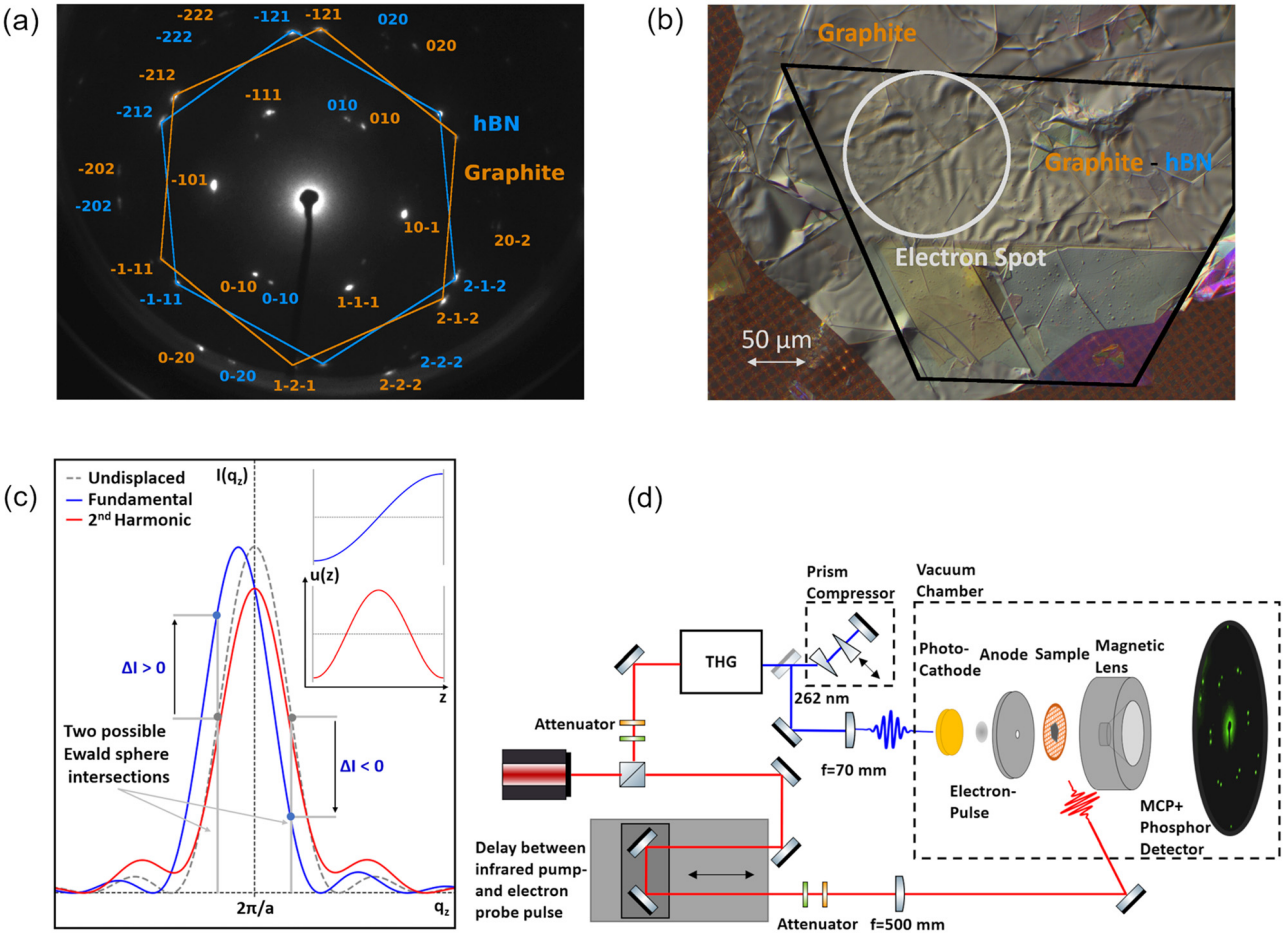
(a) Electron diffraction image of the hBN–graphite heterostructure, obtained in a sample geometry rotated by 19° with respect to the incoming electron beam. Due to the relative rotation of the hBN and graphite flakes by about 11°, the associated Bragg peaks are clearly separated despite very similar lattice constants of the two materials. (b) White-light reflection image of the hBN–graphite heterostructure, prepared on a 2000-mesh copper grid (hole width 7.5 *μ*m and hole spacing 12.5 *μ*m). The hBN film (∼20 nm thickness) on the bottom was transferred onto the grid first, followed by the graphite film on top (∼42 nm thickness). The overlap region of the hBN and graphite flake is indicated by the black outline, and the area where the electron beam probes the excited sample is marked by the white circle. For the measurement, an area with the least wrinkling was selected. (c) Schematic illustration of the reciprocal lattice rod (relrod) for the undisplaced lattice (dashed line) and the relrod modulation for Bragg orders with Miller index $\left|l\right|>0$ in the presence of the first two CAP modes in a single-material, free-standing nanofilm. The antisymmetric atomic displacement field $u\left(z\right)$ of the fundamental CAP mode (inset, blue) leads to a shift of the relrod distribution's centroid, resulting in a pronounced intensity change ΔI at the Ewald sphere intersection point. Depending on the intersection point between the Ewald sphere and the relrod, a different sign results for the intensity change. For the symmetric displacement field corresponding to the second harmonic CAP mode (red), no displacement of the relrod distribution's centroid occurs, which leads to a much less pronounced intensity modulation. (d) Schematic depiction of the experimental setup.

Our current UED setup^30,31^ is based on a Ti:Sapphire multipass amplifier with a 3-kHz repetition rate and 1.6-mJ output energy per pulse, which generates near-infrared laser pulses with 785-nm central wavelength and a pulse duration of 25-fs full width at half maximum (FWHM). A schematic illustration of the setup is shown in Fig. 1(d). Using a beam splitter, the laser beam is split into a pump and a probe beam. For electron pulse generation, the fundamental light is frequency-tripled to generate ultraviolet (UV) pulses with a central wavelength of 262 nm and 30-fs estimated pulse duration after dispersion compensation. Subsequently, the UV pulses are focused inside the ultrahigh-vacuum chamber onto a gold photocathode (40-nm gold on a sapphire substrate with a 3-nm Ti–Cr contact layer) to a focal spot diameter of about 10 *μ*m, and the fluence is adjusted to generate bunches of free electrons with 800–1200 electrons per pulse (UV pulse energies are in the range of 10 nJ). The electrons are accelerated over a 3.5-mm distance to a kinetic energy of 40 keV and traverse the specimen after an additional 5-mm field-free drift region, which yields estimated pulse durations of about 200 fs (FWHM) at the sample position. Estimation of the electron pulse duration was achieved by comparison with previous reference measurements based on grating enhanced ponderomotive scattering.^31,32^ A magnetic lens is used to focus the diffracted electrons onto the detection unit, which consists of a stack of microchannel plates (MCPs), a P43 scintillator screen, and a CCD camera to capture the image.^31^ The pump beam traverses a motorized delay stage to vary the pump-probe delay time and is then focused onto the sample via a long-focus lens (f = 500 mm). At the sample position, the beam is adjusted to a diameter of 480 *μ*m (FWHM), which ensures homogeneous excitation within the 150-*μ*m electron spot. A fluence of 1.76 mJ/cm^2^ is chosen for the experiment and corresponds to a pulse energy of 4.6 *μ*J and an estimated pulse duration below 30 fs. For each delay step, 3000 diffraction images are acquired at 200-ms exposure time and summed before evaluation to achieve a high signal-to-noise ratio. The local diffuse electron background is approximated for each Bragg spot individually by a two-dimensional plane segment in a region of interest around the Bragg spot. After background subtraction, the isolated Bragg peak of scattered electrons is fitted with a pseudo-Voigt profile^33,34^ (i.e., a weighted sum of Gaussian and Lorentzian function), and the amplitude is evaluated for each time step.

### Sample preparation

The investigated hBN–graphite heterostructure is fabricated based on the combination of mechanical exfoliation by the scotch tape method^13,14^ and a stamping method^35^ to prepare a quasi-free-standing bilayer stack on a standard 2000-mesh TEM grid. First, graphite (NGS Naturgraphit) and hBN (HQ graphene) crystals are exfoliated using commercially available transparent PDMS-based viscoelastic gel film (Gel-Pak). The exfoliated crystal flakes are examined under the optical microscope to select samples of suitable size (>50 × 50 *μ*m^2^). The flake thickness is estimated by measuring the white-light transmittance and white-light contrast in comparison with transfer matrix method calculations, taking into account the complex refractive indices of graphite, hBN, air, and PDMS. The specimen studied in this work is composed of a ∼42-nm-thin graphite layer with a lateral extent of about 0.5 mm deposited on a ∼20-nm-thin hexagonal boron nitride layer with a lateral extent of about 0.15 mm.

For transfer, a standard 2000-mesh TEM copper grid is prepared on a dome-shaped base of soluble adhesive (Crystalbond 509) by heating it on a hot plate at 90 °C for 2 min to form a thin film of adhesive that penetrates the cells of the TEM grid. After cooling down, the hBN flake is transferred onto the adhesive layer by attaching the PDMS film with the flake top-down on the mesh and applying another heating step for 30 s at 90 °C. The PDMS is then slowly peeled off, leaving the flake on the bonding layer due to the stronger adhesion. Subsequently, this step is repeated for the graphite flake after careful alignment of the two flakes under the optical microscope [see Fig. 1(a): due to the relative rotation of the hBN and graphite flakes by about 11°, the associated Bragg peaks are clearly separated despite very similar lattice constants of the two materials]. To ensure contact of the graphite crystal to the adhesive layer, the graphite flake size was chosen to exceed the lateral extent of the hBN flake. Figure 1(b) shows a reflection microscope image of the successfully transferred hBN-graphite heterostructure, with the area where the two flakes overlap marked. In a final step, the adhesive dome is embedded in filter paper and slowly dissolved by applying acetone several times, leaving a quasi-free-standing heterostructure on the TEM mesh.

## RESULTS AND DISCUSSION

To investigate the excitation and transient behavior of coherent strain waves in the hBN–graphite heterostructure, the sample is rotated by 19° with respect to the incident electron beam to achieve overlap of the Ewald sphere with (hkl) Bragg peaks with Miller index $\left|l\right|>0$. These Bragg orders are sensitive to out-of-plane atomic displacement fields^36^ associated with the longitudinal CAP breathing modes [see Fig. 1(c)]. The spectrum of photoexcited CAP modes and their phases are then encoded in the oscillatory part of the transient Bragg peak intensity, which is evaluated from the averaged data of five different Bragg spots (with either $\left|l\right|=1$ or $\left|l\right|=2$). Averaging was performed after phase matching of the extracted frequency components from different Bragg peaks. An arbitrary phase shift of 180° (corresponding to a sign change) for different Bragg peaks is related to the intersection of the Ewald sphere with the reciprocal lattice rod (relrod) profile [see Fig. 1(c)]. The relrod profile in the $q_{z}$-direction for a thin film has the character of a $\text{sin}c^{2}$ function (similar to the intensity distribution observed in the diffraction of light at the slit) and results from the coherent superposition of spherical waves of electrons scattered by the atomic lattice.

**FIG. 2.**
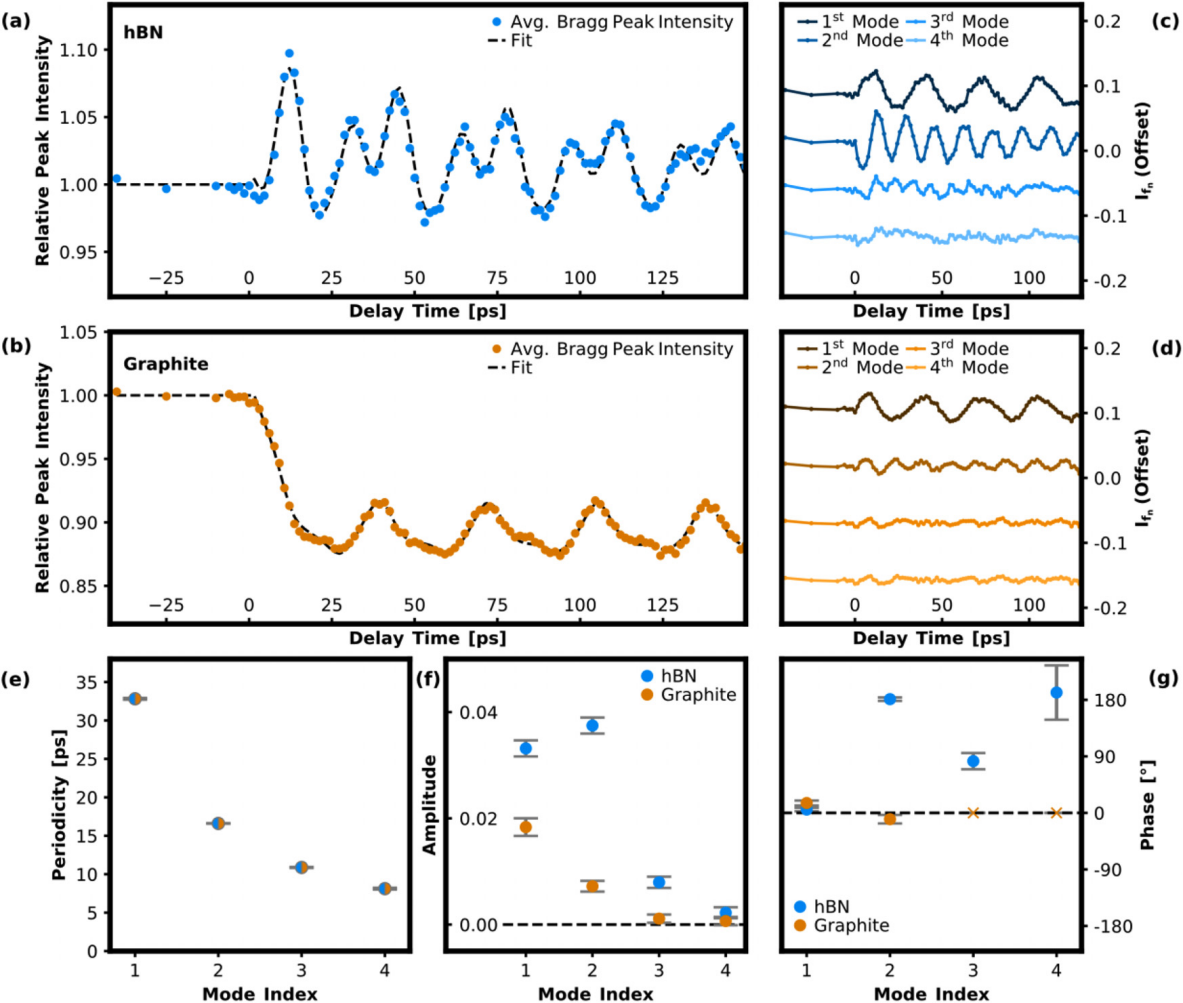
(a) and (b) Transient Bragg peak amplitude for hBN and graphite averaged over five selected Bragg peaks (with Miller index either $\left|l\right|=1$ or $\left|l\right|=2$). The data are normalized to the intensity at negative delay times before arrival of the laser pulse. (c) and (d) Extracted frequency components $I_{f_{n}}$ for hBN and graphite corresponding to the fundamental and first three higher order CAP modes. Individual frequency contributions are offset for clarity. (e)–(g) Fitted CAP periodicities, amplitudes, and phases for the fundamental and first three higher order CAP modes. Error bars show the least squares curve fitting errors. The hBN and graphite data were fitted simultaneously with the periodicities treated as a common parameter. For this reason, fitted periodicities of all four modes are indicated for both materials despite vanishing amplitudes of the third and fourth mode in graphite. In (g), phases corresponding to the third and fourth frequency component in graphite are marked with an “x,” since they have only limited significance.

Figures 2(a) and 2(b) show the transient behavior of the averaged Bragg peak intensity for graphite and hBN as a function of delay time between the laser pump pulse and the electron probe pulse. Directly after optical excitation, a multimodal oscillatory behavior can be observed in the Bragg peaks corresponding to both van der Waals layers. To extract the contained frequency components, the data were fitted with a sum of damped sinusoids superimposed with a biexponential decay to take into account the Debye–Waller effect as well as a rocking-curve contribution:^37^

$$I_{\mathrm{Fit}}\left(t\right)=\left\{\begin{array}{ll} 1, & t<t_{0}\\ 1+a_{1}\left(e^{-\frac{\left(t-t_{0}\right)}{\delta_{1}}}-1\right)+a_{2}\left(e^{-\frac{\left(t-t_{0}\right)}{\delta_{2}}}-1\right)\\ +\sum_{n}A_{n}e^{-\frac{\left(t-t_{0}\right)}{\tau_{n}}}\text{sin}(2\pi f_{n}\left(t-t_{0}\right)+\phi_{n}), & t\geq t_{0.} \end{array}\right.$$ (1)

Here, $A_{n}$, $f_{n}$, $\phi_{n},$ and $\tau_{n}$ are the amplitude, frequency, phase, and damping time of the n'th CAP mode in the bilayer stack, respectively (for values see Table I in the supplementary material),^54^ $a_{1}$, $a_{2}$ and $\delta_{1}$, $\delta_{2}$ are the amplitudes and decay time constants characterizing the biexponential decay, and $t_{0}$ is the arrival time of the laser pulse. While the Debye–Waller decay relates to the mean square atomic displacement in the heated lattice, the rocking curve contribution arises from a shift in the intersection point between the Ewald sphere and the relrod profile due to a change in the lattice constant.

The hBN and the graphite datasets were fitted simultaneously with the frequencies $f_{n}$ set as common parameters, while all other parameters are set separately for hBN and for graphite. Figures 2(c) and 2(d) show the extracted individual frequency contributions $I_{f_{n}}$ for hBN and graphite, obtained by subtracting the fitted function from the experimental data, with the respective frequency contribution of interest excluded from $I_{\mathrm{Fit}}(t)$.

Figures 2(e)–2(g) show the obtained periodicities $T_{n}=\frac{1}{{\;f}_{n}}$ as well as the mode amplitudes and phases for hBN and graphite in direct comparison. In general, the amplitudes found for the Bragg peak intensity oscillations identified with CAP modes are difficult to interpret quantitatively due to the strong dependency on the experimentally unknown intersection point of the Ewald sphere with the relrod profile.^30^ A direct inference of the atomic displacement field amplitude from the experimentally observed intensity modulation in parallel electron beam diffraction is, therefore, not possible. Nevertheless, the extracted amplitudes provide valuable information about the spectrum of CAP modes excited in the heterostructure via the photogenerated stress distribution. Furthermore, the intensities of different frequency contributions obtained for the individual material layers provide information about the relative CAP mode amplitudes, although the sensitivity of the UED technique to the corresponding displacement fields must be taken into account. The overall change of the relative peak intensity in graphite by about 10% occurring on a timescale of about 20 ps is ascribed to a superposition of the Debye–Waller effect due to the heated lattice and a shift in the Bragg scattering condition due to thermal lattice expansion. In particular, for the current pulse energies, we expect an increase in the lattice temperature of about 150 K, which would result in a Debye–Waller peak intensity drop of only a few percent for the Bragg orders considered here.^24,31^

**FIG. 3.**
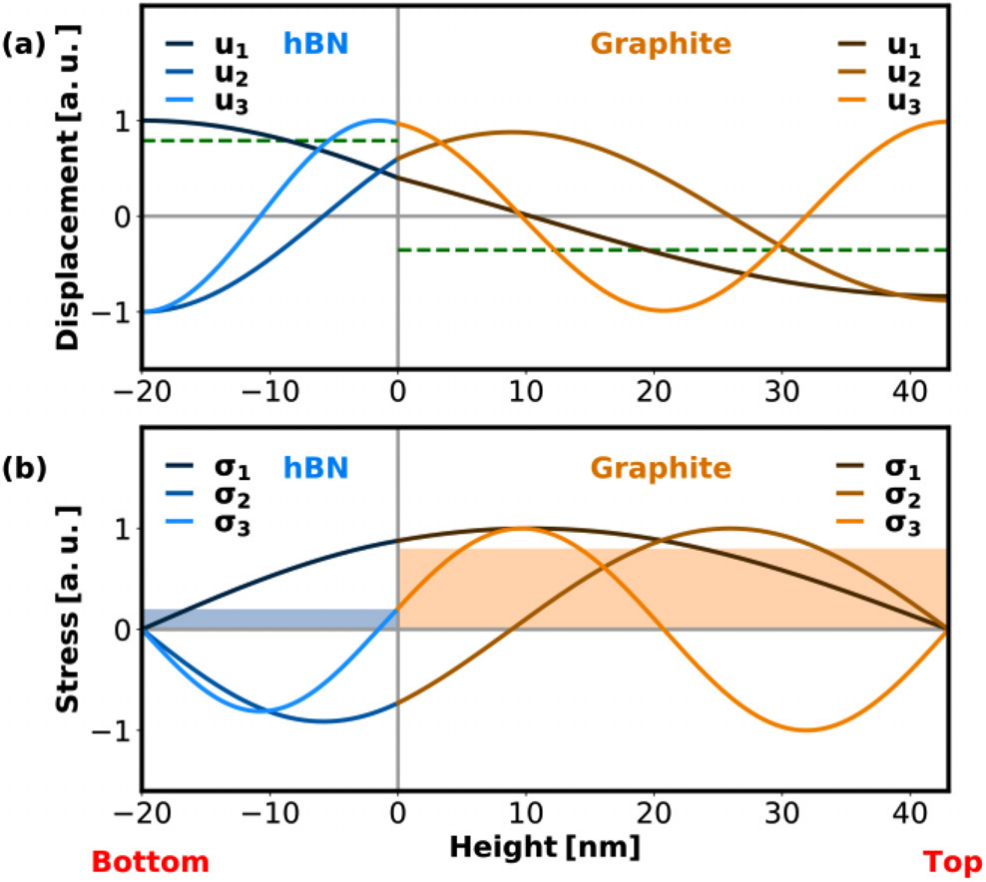
(a) Normalized atomic displacement fields for the first three CAP modes in a free-standing hBN–graphite heterostructure. The interface between the two materials is defined at height z=0 with an hBN layer thickness of 20 nm and a graphite layer thickness of 42 nm. The green dashed line indicates the mean atomic displacement of the atomic ensemble on the example of the first mode. In the analytical model, stress-free boundary conditions at the top and bottom surfaces as well as continuous displacement and stress fields at the material interface were assumed. (b) Corresponding normalized stress fields for the first three CAP modes in the heterostructure. In the background, the assumed uniform photoinduced stress distribution in the absorbing graphite layer (light orange) and a possible stress contribution in the hBN layer (light blue) are depicted.

In order to obtain a deeper understanding of the experimental observations, we employ an analytical model^38^ to calculate the resonance frequencies, out-of-plane atomic displacement fields [Fig. 3(a)], and stress fields [Fig. 3(b)] in the heterostructure for the first three resonant CAP modes at equilibrium. The material parameters for graphite and hBN used in the model are $v_{G}=4.14$ km/s and $v_{\mathrm{hBN}}=3.44$ km/s for the longitudinal out-of-plane sound velocities,^39,40^ $c_{G}=6.71\;Å$ and $c_{\mathrm{hBN}}=6.66\;Å$ for the out-of-plane lattice constants^39,40^ as well as $d_{G}=42\;\mathrm{nm}$ and $d_{\mathrm{hBN}}=20\;\mathrm{nm}$ for the film thicknesses, according to the optical characterization (see Methods section).

The photoexcited CAP modes correspond to acoustic resonances in the bilayer system described by the acoustic wave equation assuming stress-free boundary conditions at the top and bottom surfaces as well as continuous displacement and continuous stress fields at the hBN-graphite interface. Good agreement is found for the calculated periodicities of the first three CAP modes with the periodicities obtained from the experimental data [Fig. 2(e)]. We note that while the fit to the experimental data yields a non-vanishing amplitude for the fourth CAP mode (in the case of hBN, matching the periodicity obtained from the analytical model for the fourth CAP mode), it is unclear from visual inspection of the extracted trace [Fig. 2(c)] whether this mode is present in the data or not.

Interestingly, the third CAP mode is only visible in the hBN data, but not in the graphite data. An explanation for this can be derived from the calculated atomic displacement fields in the individual material layers [Fig. 3(a)]. In a UED experiment, the observed intensity for a given Bragg order corresponds to the overlap integral between the Ewald sphere and the relrod profile at their intersection point. The sensitivity of the UED technique to a specific CAP mode is, therefore, related to the change in diffracted intensity in the presence of the CAP mode's atomic displacement field. However, in the case of a bilayer heterostructure, the partial displacement fields in the respective material layers must be considered individually to describe the transient intensity modulation in the Bragg peaks.

For a discussion of the influence of atomic displacement fields on the diffraction intensity, it is helpful to distinguish between symmetric and asymmetric fields with respect to the midplane of the layer. In general, an asymmetric atomic displacement field results in a change of the mean interatomic distance in the out-of-plane direction of the thin-film. This leads to a change in the intersection with the Ewald sphere due to a shift of the relrod distribution's centroid in reciprocal space, and thus to a change in the Bragg peak intensity [see blue curve in Fig. 1(c)]. In contrast, a symmetric displacement field does not alter the average interatomic distance and, hence, will not displace the relrod distribution's centroid [see red curve in Fig. 1(c)]. Although the internal atomic shifts in the presence of a symmetric displacement field result in a minor modulation of the relrod profile as well, the change is much less pronounced compared to asymmetric fields.^30,41^ For an intuitive analysis of the experimental results, we classify the partial displacement fields of the first three CAP modes in the hBN and graphite layer as “fundamental” type (antisymmetric) and “second order” type (symmetric) in analogy to the first two CAP modes in a single-material, free-standing nanofilm. Each of the partial displacement fields corresponding to the first three CAP modes in the hBN layer can be classified as mostly fundamental type. The UED technique is, therefore, particularly sensitive to these types of modes with comparable sensitivity due to similar field distributions. The superimposed offset [as indicated by the green dashed line for the first mode in Fig. 3(a)] corresponds to a common displacement of the atomic ensemble and has no effect on the relrod distribution in reciprocal space.

For graphite, only the first two CAP modes can be categorized as mostly asymmetric fundamental type distributions, whereas the third mode is identified as a second order type distribution with an almost perfectly symmetric displacement field with respect to the midplane of the graphite layer. Therefore, a much weaker sensitivity to the third mode in graphite is expected, which explains the absence of this mode in the experimental data.

Whether a specific CAP mode in the bilayer system is excited by the laser pulse is related to the overlap integral of the stress field $\sigma_{i}(z)$ associated with the i-th CAP mode and the spatial part of the photoinduced stress distribution.^27^ In the limit of a suddenly applied stress, the weighted sum of the stress fields from all excited CAP modes is equal to the photoinduced stress distribution. In general, also the timescale of the underlying CAP excitation mechanism in relation to the periodicity of the CAP mode plays an important role, since the convolution of the transient strain impulse response with the temporal part of the photoinduced stress distribution can lead to a strong attenuation of a given mode. The two major stress contributions leading to a CAP excitation in a non-piezoelectric material are the deformation potential and thermoelastic stress, which we assume to be proportional to the spatiotemporal electronic and lattice temperature distributions $T_{e}\left(z,t\right)$ and $T_{L}\left(z,t\right)$, respectively.^29^

The experimentally observed sine-type behavior of the first and second CAP modes in both the hBN and the graphite layer [Figs. 2(c) and 2(d)], with extracted phases near 0°, suggests that the generation of these modes is associated with an impulsive excitation mechanism.^42,43^ Since hBN is a wide-bandgap semiconductor, we rule out the possibility that the electronic system in hBN is directly excited by the incident laser pulse at our excitation wavelength and fluence. Therefore, we propose that the CAP excitation in the heterostructure predominantly originates from the deformation potential within the graphite layer, with a possible additional smaller contribution from the lattice via thermoelastic stress. This assumption is also supported by the well-studied heat transfer dynamics between the electronic and the lattice system in graphite after femtosecond-laser excitation.^10,22,24^ In response to the optical pump pulse, the electronic temperature rises on a timescale of less than 150 fs,^44–46^ followed by relaxation of the hot carriers to a small subset of strongly coupled optical phonons (SCOPs) on a sub-picosecond timescale, which is short compared to the CAP periods considered here. In contrast, the subsequent heat transfer from the SCOPs to the lattice occurs on a timescale on the order of 10 ps, which is comparable to the observed CAP periodicities. We emphasize that our conclusions do not necessarily indicate that the absolute magnitude of the deformation potential in graphite dominates over thermoelastic stress, but that the excitation of the observed CAP modes with comparatively high frequencies is particularly sensitive to fast-decaying stress components.

**FIG. 4.**
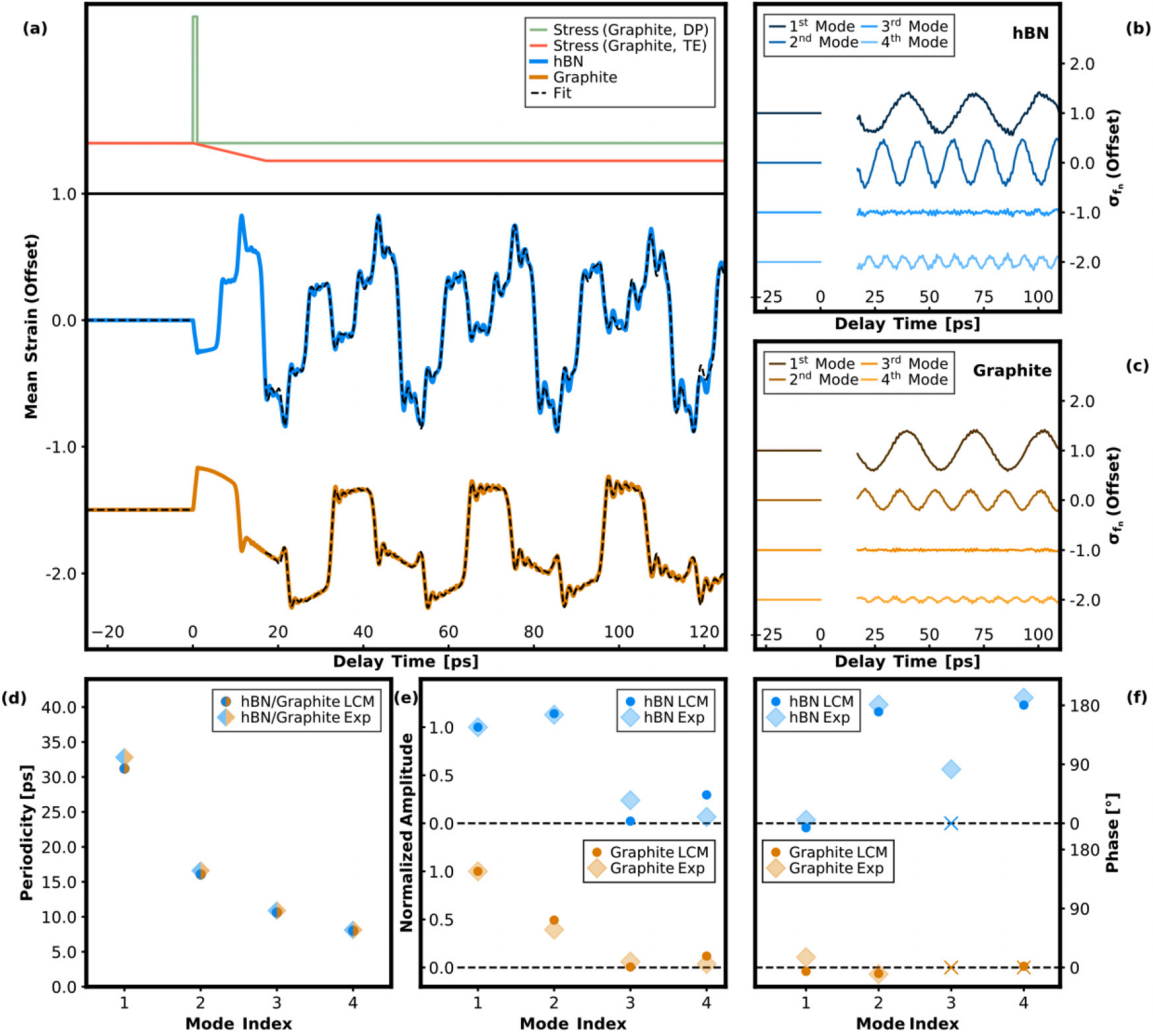
(a) Calculated transient mean strain using a linear-chain model (LCM) in the hBN layer (blue) and graphite layer (orange) assuming a spatially uniform excitation in the graphite layer. The temporal dependence of the photoinduced stress is modeled by a rectangular pulse with 1-ps duration (green) for the deformation potential (DP) (reflecting the timescale of heat transfer from hot carriers to SCOPs) and a linearly increasing stress over 17 ps (red) for thermoelastic stress (TE) (reflecting the timescale of heat transfer to the lattice). (b) and (c) Extracted frequency contributions corresponding to the fundamental and first three higher order CAP modes in hBN and graphite. Individual frequency contributions are offset for clarity. (d) and (f) Fitted periodicities, amplitudes, and phases of the frequency contributions corresponding to the fundamental and first three higher order CAP modes in hBN and graphite. In (f), phases corresponding to frequencies with an amplitude close to zero are marked with an “x,” since they have only limited significance.

In principle, we must also consider the possibility that an additional lattice-originating stress contribution arises from the SCOP system. While we cannot distinguish between a fast-decaying SCOP-related stress contribution and the deformation potential based on our data, two aspects point against a lattice origin: First, the SCOPs and the phonon modes that are populated initially via the SCOPs' primary decay channels are predominantly in-plane modes,^24,47^ which makes a significant stress contribution in the out-of-plane direction unlikely. Second, in our simulation of the strain dynamics (see below), a compressive stress with opposite sign to the thermoelastic contribution had to be assumed for the impulsive component to reproduce the experimental data correctly [Fig. 4(a)]. This out-of-plane contraction immediately after laser excitation has been observed previously in graphite and has been attributed to the electronic deformation potential.^48–50^

In order to further support the described CAP excitation model, we numerically simulate the response of the free-standing hBN–graphite heterostructure employing a one-dimensional linear-chain model^38^ and analyze the expected strain dynamics for different excitation scenarios. Following the aforementioned discussion, we neglect in our model the pronounced phonon–phonon scattering on a timescale of a few picoseconds (and possible associated stress contributions from intermediate phonon distributions) and consider only a superposition of two limiting cases: a thermal phonon bath and an impulsive excitation via the deformation potential. We assume a spatially uniform stress profile for the photoinduced stress within the graphite layer and model the temporal behavior of the impulsive component as a rectangular pulse with 1 ps duration as an approximation of the transient electronic temperature. The thermoelastic stress is modeled by a superimposed linearly increasing stress on a 17-ps timescale, and the relative thermoelastic contribution is varied in the calculation to find the best agreement with the experimental results. The 17-ps timescale used for the thermoelastic stress was derived from the slow time constant of the biexponential Debye–Waller decay in the (100) Bragg peak family measured on the same sample using a measurement geometry with the incident electron beam parallel to the sample's surface normal. We note that a comparatively wide range of values for the timescales of the heat transfer dynamics in graphite can be found in the literature.^10,24,48,49,51^ In addition to factors such as excitation fluence,^51,52^ the timescales vary depending on whether the sample is free-standing or prepared on a substrate,^53^ or whether the graphite film is embedded in a heterostructure,^25,26^ as in our case.

Figure 4(a) shows the calculated transient mean strain in the hBN and the graphite layer under the aforementioned conditions. We chose the mean strain in the hBN and the graphite layer as a suitable quantity for comparison with the experimentally obtained intensity modulation since it directly relates to the change in mean interatomic distance (see Discussion earlier). We note, however, that the absence of very high-frequency components in the experimental data present in the calculated mean strain is likely due to the more complex relationship between the intensity modulation caused by higher order CAP modes and the associated modulation of the mean strain.^30^ Individual frequency contributions contained in the calculated transient for the first four CAP modes are obtained in the same way as described for the experimental data by subtracting the fit with the frequency of interest excluded. The analysis of the extracted frequency components [Figs. 4(b) and 4(c)], fitted amplitudes, and phases [Figs. 4(e) and 4(f)] reveals that the experimental observations are essentially well-captured under the assumption of this excitation scheme. A relative thermoelastic stress contribution with a magnitude of 14% of the peak of the deformation potential yielded the best agreement with the experimental data. In particular, the relative amplitudes of the first and second mode are well reproduced for both materials, and the extracted phases are in good agreement as well.

Interesting to note is the phase shift of the second mode in hBN by 180° compared to graphite, which is also observed in the experiment. From the calculated partial displacement field and derived stress field of this mode in hBN and graphite (see Fig. 3), one can see that this phase shift is related to the opposite sign of the mean stress in the hBN and the graphite layer: an expansion of the hBN layer causes a compression of the graphite layer and vice versa.

An important discrepancy to the experimental findings must be pointed out, namely, that the third mode is not excited using this excitation model, and the corresponding frequency component is neither contained in the calculated hBN nor in the graphite data. However, upon closer examination of the partial stress fields corresponding to the third CAP mode [Fig. 3(b)], this is to be expected. Since the excited amplitude of a specific CAP mode is proportional to the overlap integral of the associated stress field with the photoinduced stress distribution in the bilayer system, this integral vanishes for the nearly antisymmetric partial stress profile of the third CAP mode in graphite, assuming uniform excitation in the graphite layer.

To consider the possibility that thermoelastic stress plays the dominant role in the CAP excitation process, we also calculated the mean strain dynamics in a purely thermoelastic excitation scenario (for detailed evaluation, see supplementary material,^54^ Fig. 1). Here, it is found that the experimentally observed relative amplitudes of the first and second CAP modes in hBN and graphite are not reproduced well. Due to the significantly longer excitation time, which is comparable to the periodicities of the CAP modes considered here, the second mode is strongly attenuated compared to the fundamental. Furthermore, the phases extracted from the calculation (close to ±90° for the second mode) do not match the experimentally determined values for this excitation scheme.

Hence, given the good agreement of the experimental results with the first excitation scenario, we conclude that thermoelastic stress in the graphite layer plays only a minor role for the CAP excitation in the investigated heterostructure. We note, however, that this conclusion may not apply to thicker layers with CAP periodicities that are long compared to the timescale of thermoelastic stress generation.^41^

Finally, addressing the origin of the experimentally observed third CAP mode in the hBN dataset, we consider the possibility of a thermoelastic stress contribution originating from the hBN layer in addition to the stress components in graphite. This assumption is based upon reports that indicate an ultrafast heat transfer mechanism between the graphite and the hBN layer in hBN-graphite heterostructures, which has been attributed to either the coupling of hot carriers or SCOPs in graphite to hyperbolic phonon polaritons in hBN.^25,26^ The associated generation of thermoelastic stress in the hBN layer then leads to a combined excitation model with the detailed numerical analysis shown in the supplementary material^54^ (Fig. 2). It can be seen that the excitation of the third mode is reproduced here, with the extracted phase (close to 90°) in good agreement with the experimental data for hBN. For the model, a relative thermoelastic stress contribution in hBN with a magnitude of 10% of the peak of the deformation potential in graphite was chosen to achieve good agreement with the experimental results. It should finally be noted that a non-uniform stress profile in the graphite layer could also be considered as an alternative explanation for the excitation of the third mode, although, in this case, our numerical analysis yields a phase that does not match the experimentally obtained phase.

## CONCLUSION

We investigated femtosecond-laser-excited coherent strain waves in a van der Waals heterostructure comprised of a ∼42-nm graphite layer stacked upon a ∼20-nm hBN layer. The multimode oscillatory behavior observed in the Bragg peaks of both materials is identified with resonant CAP modes that are distributed along the system's out-of-plane direction. Frequency contributions extracted from the experimental data are analyzed and compared with the acoustic resonance frequencies obtained from an analytical model. Taking into account the atomic displacement fields that correspond to the observed CAP modes, the sensitivity of the UED measurement technique to partial fields in the respective material layers is discussed. Extracted frequency contributions are analyzed in terms of amplitudes and phases to draw conclusions about the dominant CAP excitation mechanism present in the heterostructure. Since only the graphite layer is accessible to direct excitation by the laser pulse due to our choice of materials, the question arises which CAP excitation mechanism is acting in the combined material system. To further address this question, we used a numerical linear-chain model to compare different excitation scenarios. Here, we identified the deformation potential in graphite as the main excitation mechanism in the heterostructure, with an additional minor thermoelastic stress contribution. Furthermore, the contribution of a superimposed thermoelastic stress from the optically transparent hBN layer is included in the model, which hints toward an ultrafast heat transfer mechanism from graphite to the hBN lattice system. Our results, thus, not only contribute to the understanding of excitation and dynamics of coherent acoustic phonons in van der Waals heterostructures, but also shed light on the interactions between the materials involved. These couplings have to be considered when employing such heterostructures in devices. Moreover, our results experimentally demonstrate an intriguing possibility to tailor the excitation of specific CAP modes in single-sided excited heterostructures selectively, by adjusting partial strain fields via tuning of the thickness ratio.

## Data Availability

The data that support the findings of this study are available from the corresponding author upon reasonable request.
